# Metastatic Urothelial Cancer Presenting as Small Bowel Obstruction: A Case Report

**DOI:** 10.7759/cureus.61228

**Published:** 2024-05-28

**Authors:** Ahmed Alsaiari

**Affiliations:** 1 Department of Internal Medicine, University of Jeddah, Jeddah, SAU; 2 Department of Gastroenterology, MedStar Georgetown University Hospital, Washington, USA

**Keywords:** upper git surgery, endoscopic ultrasound (eus), git endoscopy, metastatic urothelial carcinoma, bowel obstruction

## Abstract

Neoplasms are among the common causes of small bowel obstruction (SBO). Metastatic disease is the most common cause of neoplastic SBO and is most commonly the result of colon, ovarian, pancreatic, and gastric neoplasms. Metastatic SBO secondary to metastatic urothelial carcinoma is exceedingly rare, with only a few cases described in the literature. It is important for physicians to be aware of urothelial carcinoma as a potential etiology of SBO.

## Introduction

Acute mechanical small bowel obstruction (SBO) accounts for approximately 2% to 4% of emergency department visits, 15% of hospital admissions for abdominal pain, and 20% of emergency surgical operations for abdominal pain [1,2]. While SBO is most commonly caused by adhesive disease following abdominal surgery (55%-80%), the second most common cause is neoplasm (20%) [3,4]. While primary tumors can cause SBO, the majority of neoplastic SBO is caused by metastatic disease. Malignancies can cause SBO by either external compression of the bowel or by endoluminal obstruction. Malignancies with a propensity to cause widespread peritoneal metastases, such as colon, ovarian, pancreatic, and gastric neoplasms, are known to be the most common cause of malignant SBO due to external compression of the bowel. Tumors that spread hematogenously, such as melanoma, lung, breast, cervix, colon, and various sarcomas, can involve the wall of the small bowel and cause endoluminal obstruction.

Bladder cancer is the most common malignancy of the urinary tract, with urothelial cancer making up approximately 90% of all bladder cancers [5]. While urothelial cancer is known to metastasize to lymph nodes, bones, lungs, liver, and peritoneum, it is an extremely rare cause of SBO, with only a handful of cases described in the literature to date [6-9]. In this case report, we present a case of metastatic duodenal SBO secondary to metastatic urothelial carcinoma.

## Case presentation

**Figure 1:**
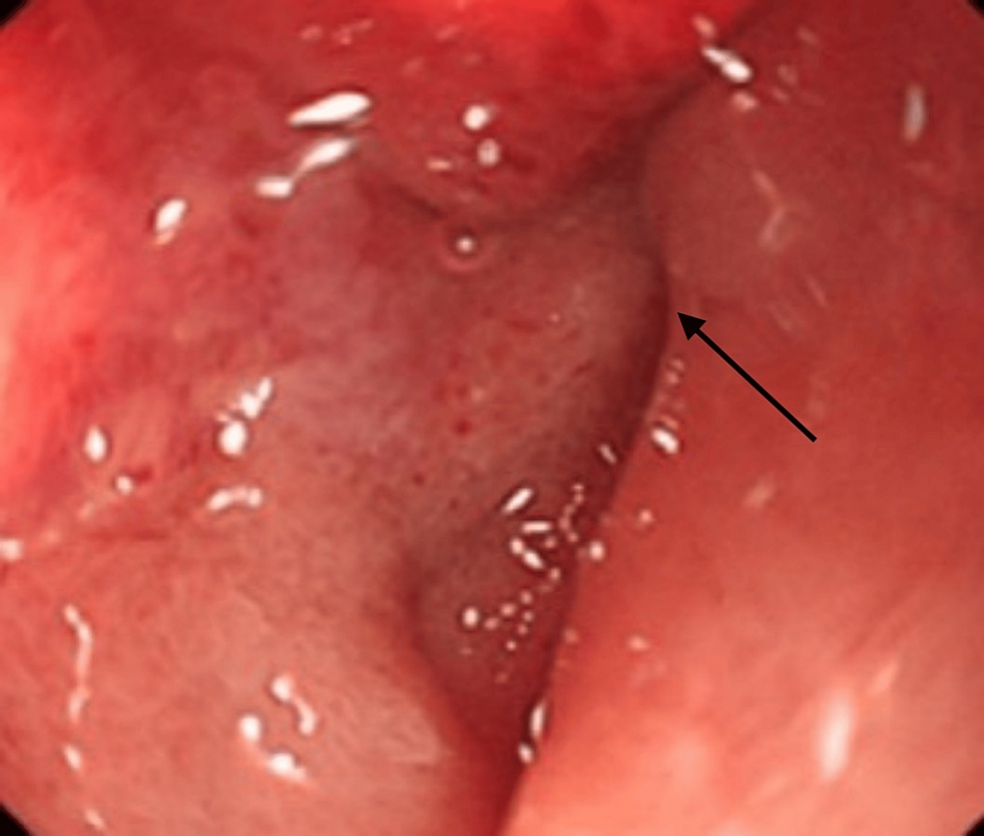
EGD image showing acute malignant obstruction of the second portion of the duodenum (arrow) secondary to external compression from metastatic urothelial carcinoma. The endoscope was unable to be passed through the stricture. EGD, esophagogastroduodenoscopy

A 71-year-old male presented to the emergency room of an outside institution with a chief complaint of nausea, vomiting, and epigastric pain following solid oral intake, along with one episode of dark red emesis the day before the presentation. Of note, the patient had a history of multiple perforated duodenal ulcers requiring surgical repair in 2016 and 2021, as well as a history of urothelial bladder carcinoma (status post cystectomy with ileal conduit and right lower quadrant urostomy in 2014). Physical exam on admission revealed a non-toxic-appearing elderly male with a mildly distended abdomen with tenderness to palpation in the epigastric region, normal bowel sounds, no peritoneal signs, and normally draining urostomy. Lab workup was significant for leukocytosis to 16.0 with neutrophilic predominance but was otherwise unremarkable. Computed tomography (CT) of the abdomen with intravenous contrast showed marked gastric distention without a visualized obstructing lesion, as well as retroperitoneal adenopathy concerning for recurrence of urothelial carcinoma. Esophagogastroduodenoscopy (EGD) at this time showed a narrowing of the descending duodenum with an inability to pass the endoscope through the second part of the duodenum (Figure 1). A nasogastric tube was successfully passed for gastric decompressions. The patient was transferred for further investigation of his duodenal obstruction. 

**Figure 2:**
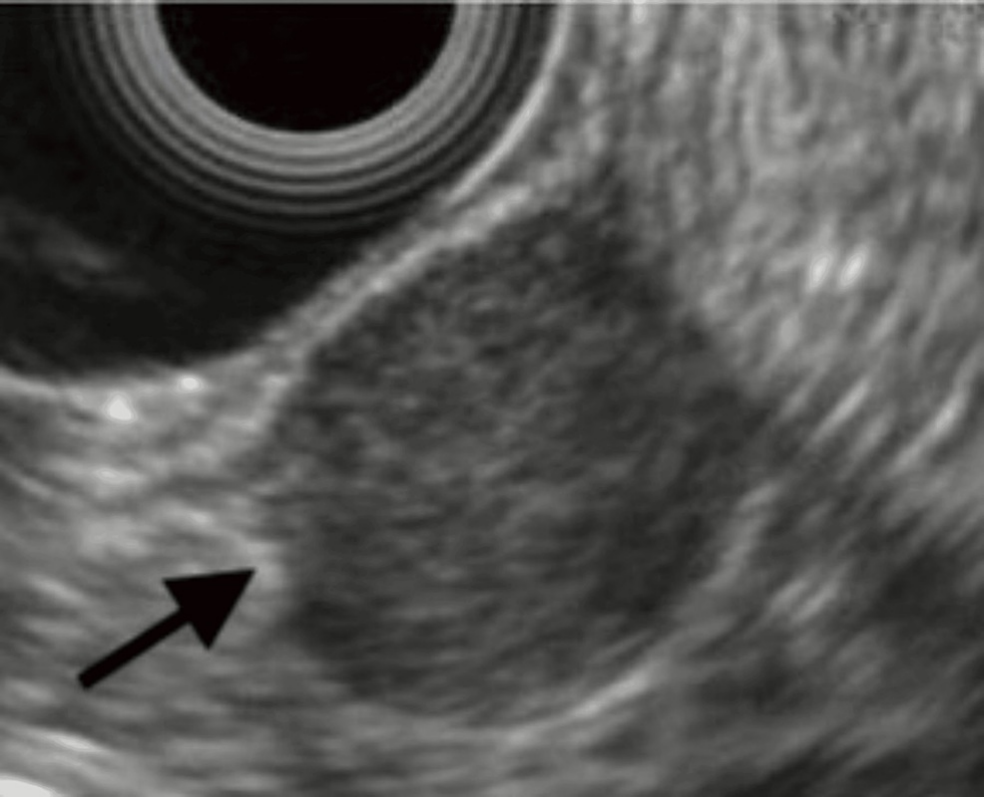
EUS image showing an 18.2 mm x 17.9 mm hypoechoic lesion at the level of first portion duodenal structure arising from the muscularis propria of the duodenal wall (arrowed). FNA biopsy of the lesion confirmed the recurrence of the patient’s urothelial carcinoma. EUS, endoscopic ultrasound; FNA, fine-needle aspiration

The patient underwent further investigation with endoscopic ultrasound (EUS), which showed an 18.2 mm x 17.9 mm hypoechoic lesion at the level of the first portion duodenal structure arising from the muscularis propria of the duodenal wall (Figure 2). Fine-needle aspiration (FNA) biopsy was performed, which confirmed the recurrence of his urothelial carcinoma. The patient also underwent a positron-emission tomography (PET) scan, which revealed a hypermetabolic duodenal mass, in addition to extensive retroperitoneal lymphadenopathy. Based on the patient’s goals of care preferences, he was initiated on total parenteral nutrition (TPN), and a venting gastrostomy tube (G-tube) was placed for gastric decompression. He was initiated on cisplatin-based chemotherapy during his hospitalization, with plans to discontinue TPN and convert his G-tube to a feeding gastrojejunal (GJ) tube following the resolution of his malignant obstruction.

## Discussion

Physicians need to recognize urothelial carcinoma as a rare but potential cause of acute SBO secondary to metastatic malignancy, particularly in patients with a history of urothelial carcinoma. While it is most common for urothelial carcinoma to metastasize to lymph nodes, bones, lungs, liver, and peritoneum, it has rarely been described to metastasize directly to the muscular wall of the duodenum [8,10-13]. In cases where malignant SBO is suspected, patients must undergo EGD for direct visualization of the gastrointestinal tract and biopsy, by either direct mucosal biopsy when possible or EUS with FNA biopsy when the lesion is not directly visualized in the intestinal mucosa.

It is essential to work through a full differential diagnosis when presented with a case of SBO. The most common cause of SBO is adhesive disease (55%-80%) and should be considered in all patients with a history of abdominal surgery, including this patient, who had a history of perforated duodenal ulcer twice repaired with surgery [4]. Malignancy is the second most common cause of SBO (20%) [3]. Other common causes of SBO include Crohn's disease (3%-7%), volvulus (4%-15%), intussusception (4%-8%), and gallstones (2%) [14,15]. Physical exam findings in a patient with SBO will most commonly show abdominal distention (more common in distal obstructions) and either hyperactive (early) or hypoactive (late) bowel sounds. The initial workup should include basic lab work and abdominal imaging. The test of choice for the initial evaluation of SBO is usually an abdominal CT scan with intravenous (IV) contrast, given its superior sensitivity (over 96%) and specificity (up to 100%), as well as the ability to assess for intestinal ischemia [16]. Imaging modalities may also include abdominal X-ray (sensitivity 66%-77% and specificity 50%-57%), small bowel series (sensitivity 97% and specificity 96%), and point-of-care ultrasound (sensitivity 97.7% and specificity 92.7%) [17-19]. In patients who are acutely ill, who have peritoneal signs, or who have signs of current or impending bowel strangulation on imaging, surgery should be consulted for urgent operative evaluation. In cases where urgent surgery is not indicated and imaging is nondiagnostic, EGD should be performed to directly visualize the intestinal mucosa and obtain biopsies, if warranted. 

In cases where malignant SBO is confirmed, a multidisciplinary approach involving gastroenterology, medicine, surgery, and medical oncology is necessary. In most cases, gastric decompression via NG tube or vented G-tube can lead to symptomatic relief. The patient should be treated symptomatically for nausea, vomiting, and abdominal pain while the obstruction persists. Medical oncology should be consulted for the evaluation of chemotherapeutic treatment options. In most cases of urothelial carcinoma, cisplatin-based chemotherapy is the first-line therapy and can lead to decreased tumor burden and possible relief of the obstruction [20]. It is important to clarify medical goals of care with patients early in the treatment process, particularly regarding nutritional therapy. In patients where it fits with their goals of treatment, it may be beneficial to initiate TPN or tube feeding until the obstruction is resolved and the patient can be transitioned back to oral intake.

## Conclusions

We present a case of metastatic duodenal SBO secondary to metastatic urothelial carcinoma. Physicians need to recognize urothelial carcinoma as a rare but possible cause of acute malignant small bowel obstruction. Management of acute malignant SBO requires a multidisciplinary approach between gastroenterology, medicine, surgery, and medical oncology and should involve goals of care discussions with patients regarding the appropriate treatment and feeding strategy.
